# Gene Expression and Regulation of Higher Plants Under Soil Water Stress

**DOI:** 10.2174/138920209788488535

**Published:** 2009-06

**Authors:** Fu-Tai Ni, Li-Ye Chu, Hong-Bo Shao, Zeng-Hui Liu

**Affiliations:** 1 1College of Life Sciences, Jilin Normal University, Siping 136000, China; 2 2State Key Laboratory of Soil Erosion and Dryland Farming on the Loess Plateau, Institute of Soil and Water Conservation, Chinese Academy of Sciences, Yangling 712100, China; 3 3Shandong Key Laboratory of Eco-environmental Science for Yellow River Delta, Binzhou University, Binzhou 256603, China; 4 Institute of Life Sciences, Qingdao University of Science & Technology, Qingdao 266042, China

**Keywords:** Higher plants, soil water stress, gene regulatory network, drought, anti-drought gene resources, signal, ion homeostasis, physiological mechanisms.

## Abstract

Higher plants not only provide human beings renewable food, building materials and energy, but also play the most important role in keeping a stable environment on earth. Plants differ from animals in many aspects, but the important is that plants are more easily influenced by environment than animals. Plants have a series of fine mechanisms for responding to environmental changes, which has been established during their long-period evolution and artificial domestication. The machinery related to molecular biology is the most important basis. The elucidation of it will extremely and purposefully promote the sustainable utilization of plant resources and make the best use of its current potential under different scales. This molecular mechanism at least includes drought signal recognition (input), signal transduction (many cascade biochemical reactions are involved in this process), signal output, signal responses and phenotype realization, which is a multi-dimension network system and contains many levels of gene expression and regulation. We will focus on the physiological and molecular adaptive machinery of plants under soil water stress and draw a possible blueprint for it. Meanwhile, the issues and perspectives are also discussed. We conclude that biological measures is the basic solution to solving various types of issues in relation to sustainable development and the plant measures is the eventual way.

## 1. INTRODUCTION

Environmental stresses represent limiting factors for plant productivity on the globe. Drought is one of the important abiotic stresses, constraining global crop production and quality seriously and recent global climate change and increasingly erratic weather patterns in the future are likely to enhance this situation more seriously [1-11]. Abiotic stress factors mainly include temperature, salinity, drought, anaerobic, and mechanical stresses on plants. In most cases, soil water deficits directly result in drought, which is closely linked with natural rainfall [12-21]. Drought is a complex physical-chemical process, in which many biological macromolecules and small molecules are involved ,such as nucleic acids (DNA, RNA, microRNA), proteins, carbohydrates, lipids, hormones, ions, free radicals ,mineral elements [22-78]. In addition, drought is also related to salt stress, cold stress, high temperature stress, acid stress, alkaline stress, pathological reactions, senescence, growth, development, cell circle, UV-B damage, wounding, embryogenesis, flowering, signal transduction and so on [79-100], making the problem more complicated. The development and application of modern molecular biology have led to a better understanding of plant adaptations and responses to abiotic stress conditions. Genes responsible for adaptation processes to every example of given stress have been identified in Arabidopsis thaliana and other important model plants. The use of transgenic plants to overexpress or silence these genes is a powerful tool in determining if they are necessary or sufficient to induce stress tolerance. However, with stress tolerance being a quantitative character, it should not be expected that single genes confer a high level of corresponding tolerance [101-115].

Considering the interacting complexity (at least including water movement, solute transport, information exchange, ion homeostasis regulation, and other related physi-chemical changes) between plants and their surroundings, it is necessary to generalize the performance of physiological functions for higher plants under drought stress. In this article, related aspects of physiological and molecular responses of higher plants to drought stresses will be reviewed, mainly including the following parts: Plant Physiological Function Performance under Soil Water Stress, Plant Gene Regulatory Network System, A Model for Stress Signal Transduction Pathway in Higher Plants under abiotic Stresses, Pant Gene Regulatory Network System and Plant Drought Resistance Improvement. We then focus on the aspects of plant gene regulatory network system, which is the core controlling the interrelationship between plants and environment at the molecular level in a complex and coordinated manner.

## 2. PLANT PHYSIOLOGICAL FUNCTION PERFORMANCE IN FIELD UNDER SOIL WATER STRESS

Plants live in soil-plant-atmosphere continuum (SPAC) environment, and they have to coordinate the mechanisms of diverse types to respond to the above changing environment at any time for sustainable survival [1-9]. When abiotic-stress happened, the water in plants would be distributed again. And it could make the old leaf dead by the water movement from old leaf to new leaf, resulting in reduction of the yield of crops. Plant productivity realization is obtained eventually through physiological pathways at least at the level of individual and community [10-16]. One molecule, one kind of tissue or an organ can not produce any economic yield in terms of the need for human being [26-53]. Under the condition of ensuring plant survival, plants can produce corresponding yield.

Soil water is one of key factors influencing plant production and many reports have proved this clearly [17-25]. Loss of water in soil will lead to great reduction in plant production, which has been reflected from total grain yield of many countries in the world [54-58]. Soil water is also the important material for photosynthetic reactions that plants depend on to finish accumulation of photosynthetic products, which are impacted greatly by physiological pathways and environmental factors (such as soil water supply) [59-64]. The influence of water deficits for plant metabolism is very apparent, which is mainly restraining the anabolism by reducing the activity of synthase and strengthening the catabolism by increasing the activity of hydrolytic enzymes. This includes the reduction of protein, chlorophyll, DNA, RNA and plant growth hormone synthesis, which could destroy the normal metabolism and cause growth disorder. So, different soil water supplying will result in quite different physiological pathways, which directly determine the ability for plants to make photosynthetic products. Water deficits in soil environment also influence solute transport (ion and nutrient uptake of plants) to larger extent, which effects on photosynthetic reactions in plant chloroplasts in many ways [65-71]. This is the reason that ion homeostasis and redox state have been brought to attention [72-76].

The series of the above reactions and processes occurring at different biointerfaces is regulated and controlled by plant gene regulatory network system spatially and temporally on the basis of responding to plant developmental cue, through which plants can elegantly respond to the changing environment [77-82]. This network system has been formed by the interaction between plants and environment for a long time of evolution, which will continue to evolve with environmental succession [83-86]. From the angle of individual plant development, Plant Growth Grand Periodicity curve can reflect and show the above trend, displaying higher plasticity [87-90]. Besides, plant responses to soil water deficits (including nutrients) take a “slow-fast-slow” shaped curve in terms of main physio-biochemical indices change and this is in agreement with Plant Growth Grand Periodicity, which also illustrates this fact and wide plasticity for plants [36-53]. Surely, concerted expression of corresponding genes in plant gene regulatory network system makes it possible that we can see the phenotype and phenotype change under given temporal-spatial condition [91-96].

## 3. PLANT GENE REGULATORY NETWORK SYSTEM UNDER ABIOTIC STRESSES

Recent progress in molecular biology and bioinformatics (especially, DNA microarray technology), genomics, proteomics, metabolomics and transcriptomics) has provided insight into plant gene regulatory network system, which is mainly composed of inducible-genes (environmental factors and developmental cues), their expression programming and regulatory elements (cis-element and trans-element), corresponding biochemical pathways and diverse signal factors [97-103]. The genetic information for drought tolerance is expressed in many prokaryotes and lower eukaryotes, but only in very few higher plants. In higher plants, only seeds can survive for extended periods without water. Exceptional among higher plants is the small group of angiosperm plants termed 'resurrection plants' which can recover from complete dryness within one day of contact with water [116]. Under the condition of soil water deficits, related stress factors always result in overlapping responses, including anatomical, physiological, biochemical, molecular biological changes, which make plant gene regulatory network system more complicated and difficult to explore. Much information with respect to this topic is from the model plant, *Arabidopsis thaliana*. Main aspects will be illustrated below.

### 3.1. Typical Environmental Stress-responsive Transcriptional Elements

Plants can sense, process, respond to environmental stress and activate related-gene expression toincrease their resistance to stress. Environmental stress-inducible genes can be mainly divided into two types in terms of their protein products: one type of genes, whose coding products directly confer the function of plant cells to resist to environmental stress such as LEA protein, antifreezing protein, osmotic regulatory protein, enzymes for synthesizing betaine, proline and other osmoregulators; the other type of genes, whose coding products play an important role in regulating gene expression and signal transduction such as the transcriptional elements for sensing and transducing the protein kinases of MAP and CDP, bZIP, MYB and others [103-105]. Though these stress genes could be induced, they have not tissue specificity. At the same time, the protein of most different genes coding drought-resistance is rich in Gly, Pro and other hydrophilic amino acids, but the content of Trp and Cys is lower than others. Li *et al.* (1997) found that the content of Pro was 38 percentage in the 41.5kD drought-induced-protein in wheat seedlings, but it had not Trp and Cys [117].

Transcriptional elements are defined as the protein combining with the specialized DNA sequence of eukaryotic promoters or the protein having structural characteristics of known DNA-combining region, whose main function is to activate or suppress transcriptional effect of corresponding genes [61-69]. It is a critical factor for the transduction of stress signal. The transcriptional elements would be synthesized under the stress, and it could deliver and amplify the signal to regulate the genes expression to change the physiological function performance. There are two kinds of transcriptional elements according to their characters of expression: first, constitutive transcription elements, which could express normally in stress such as HvCBF2 in barley [118, 119]; the other is an inducible transcription element, whose expression mainly occurs in stress environment. Most transcription elements are the second type. It is proved that there are four function domains by protein analysis: DNA-binding domain, transcriptional regulatory domain (including activation and inhibitory domains), oligomerization site and nuclear localization signal. These domains can regulate the gene expression with the function domains of other transcriptional elements or promoter cis-acting elements in specific time [120]. But there are not sufficient evidences to prove how these transcription elements regulate the target gene expression and depict a legible stress-signal-responsive system.

Up to now, hundreds of transcriptional elements of environmental stress-responsive genes in higher plants have been isolated, which regulate and control the stress reaction related to drought, salinity, cold, pathogen and heat. In the genome of *Arabidopsis *and rice, they have about 1300-1500 genes for coding transcriptional elements, most of which have not been identified functionally. Recent study has shown that the transcriptional elements involved in plant stress responses mainly include four kinds: APETALA2/ EREBP, bZIP, WRKY, and MYB [70-79]. Some typical plant transcriptional elements have been summarized in Table **1** for reference.

### 3.2. Complexity of Plant Gene Regulatory Network System: Specificity and Crosstalk

Many transcriptional element families participate in plant stress responses, each of which has many members with highly-conservative DNA-binding domain, composing a complicated, temporal-spatial network system for plant gene expression and regulation [81-86]. The specific amino acid sequence of DNA-binding domain decides the specificity of distinguishing and combining with cis-acting elements. Different members of TGA/OBF families have different DNA-binding specificity, protein-protein interaction and expressing profiles. Chromatin immunoprecipitation techniques indicated that tobacco TGA1a *in vivo* combined with xenobiotic-responsive promoters, but could not combine with PR promoter with as-1 cis-element [103-105]. Arabidopsis TGA2 could be responsive to SA signal, but not be responsive to xenobiotic stress signals.

Much analysis of genomic expression profiling by DNA microarray indicates that the mRNA coding transcriptional element genes in many plants are usually induced to express and accumulated [91, 98]. Most transcriptional element genes involved in plant stress responses have not only completely different expression profiles, but also some overlapping expression profiles, showing the complexity, specificity and crosstalk of plant gene regulatory network system [58-63]. In other words, one kind of stress may simultaneously activate many transcriptional elements and one transcriptional element may be activated by many types of plant stress responses. For instance, CBF3/DREB1a can be responsive rapidly to cold, at the same time, regulated by circadian clock [71-78], which reflects the functional complement between plant cold-responsive pathway and circadian clock-regulated circle in terms of CBF3/DREB1a functions [36, 54, 88, 94-105]. The gene, SbPRP derived from 2-week-old soybean seedlings, encodes 126 amino acids, which have a signal peptide in N-terminal. It is mainly in leaf and epicotyl, and its transcription is regulated by water-stress, salt-stress and plant hormone at the same time [121].

Shinozaki *et al*. (2003) thought that four signal pathways regulating the gene expression were involved in plant drought, cold and salinity responses, in which two were ABA-dependent (I and II), and two were non-ABA-dependent (III and IV). The gene expression depends on ABA accumulation in plants, which would change with the content of ABA. There are some conservative ABA responsive elements in these genes, whose characteristic sequence is PyACGTG (G/T)C. It can regulate the stress gene expression induced by ABA. Yamaguchi-Shinozaki *et al.* (1989) found two conservative cis-acting elements, motif 1(GTACGTGGC) and motif 2(CGG/CCGCGCT) after comparing four rab16 gene promoters of rice. Through analysis they found that motif 1 is the cis-acting factor to ABA reaction [122-125]. The gene expression of non-ABA-dependent pathway would be affected by drought and cold beside ABA. It means that ABA is unnecessary for its expression. Some studies indicated that it had a DREB transcriptional element in non-ABA-dependent pathway, which can distinguish the DRE element in the gene promoter. DRE/CRT is a cis-acting element in higher plants to respond the drought and cold stress, whose characteristic sequence is CCGAC. There are many elements associated with DRE found now. It is proved that the involvement of these elements in the stress response is without depending on ABA-dependent gene expression [126-129].

The process of stress signal sensing and transducing, transcriptional regulating, and functional expressing was existent in these pathways [106-115]. Zhu T (2003) and Zhu JK (2000, 2003) concluded that molecular mechanism of plant stress responses (drought and salinity) included three main steps, i.e. stress signal input, transducing process, and regulatory product output through the study of *Arabidopsis *drought and salinity for many years. Results of many genetic mutants and key intermediate molecules from his lab supported his view powerfully. Recent related anti-drought data (dynamic change of anti-oxidative enzymes and soil water stress threshold) from my lab also proved the point [58-66]. From plant developmental context, plant responses to environmental stresses have a universal law, which has been reflected completely by Plant Growth Grand Periodicity curve. Our study on dynamic changing of wheat anti-oxidative enzymes under soil water deficit have indicated that wheat with different genotypes responded to soil water stress by taking a“ slow- rapid- slow” characteristic curve during wheat life cycle. This is the physiological basis for water-saving agriculture and dry land farming, which also provides substantial evidence for the above viewpoint [34, 37, 46, 49, 50, 91, 95, 103, 105-115].

## 4. A POSSIBLE MODEL FOR STRESS SIGNAL TRANSDUCTION PATHWAY IN HIGHER PLANTS UNDER ABIOTIC STRESSES

Animals must change their behavior to fit the living environment fluctuates. The same case happens on higher plants and sessile higher plants must also change behavior to increase fitness as the local environment fluctuates. A stronger spatial dimension network underlies signal transduction; for instance, and higher plants must be able to detect gradients in signals (such as light) and resources (such as nitrate and water). Higher plant development itself also is decidedly polar [7-11]. The spatial dimension is satisfied in many ways. Higher plant cells place receptors, channels, G proteins, and kinases, in specific membranes. Some signaling protein complexes are permanent, such as relatively stable and perhaps hardwired COP9 signalosome. Other signaling protein complexes are likely to be ephemeral and formed immediately as a result of signaling [12-16]. There are at least 300 receptor kinases in Arabidopsis, and most of them are membrane bound. Incompatibility and disease defense signal transduction use receptor kinases. After ligand binding and autophosphorylation, such kinases may act as nucleation sites for the construction of ephemeral signaling complexes that contain many proteins [21-25].

**Fig. (1):**
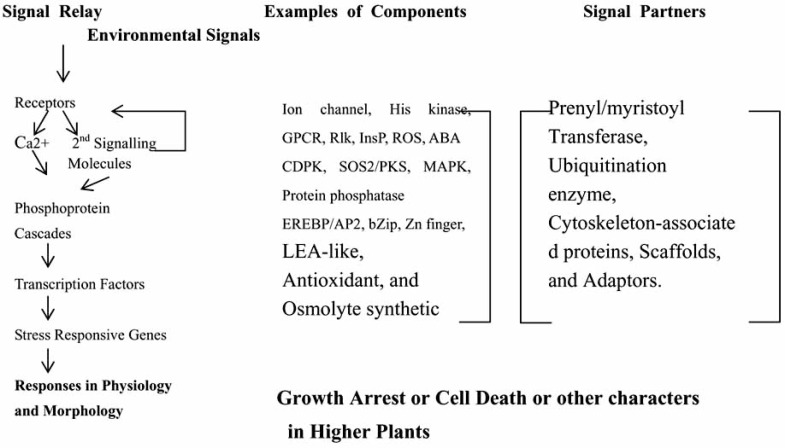
A framework for the signal transduction of abiotic stresses in higher plants.

Although there are some differences in different higher plants, a common signal model for stress transduction pathway exist in higher plants [104-115] (Fig.**1**). This model begins with the perception of signals from environments, followed by the generation of second messengers (such as inositol phosphates and reactive oxygen species). Second messnengers can modulate intracellular Ca2+ levels, often initiating a protein phosphorylation cascade that finally targets proteins directly involved in cellular protection or transcription factors controlling specific sets of stress-regulated genes. The products of these genes may participate in the production of regulatory molecules like the plant hormones abscisic acid (ABA), ethylene, and salicylic acid (SA). Some of these regulatory molecules can, in turn, initiate a second round of circulation.

## 5. PANT GENE REGULATORY NETWORK SYSTEM AND PLANT DROUGHT RESISTANCE IMPROVEMENT

Pant Gene Regulatory Network System is very complex. More information is obtained from the model plant, *Arabidopsis thaliana*. The transcriptional elements induced by stress signals decide which gene expression should be increased and the function of different transcriptional elements in plants is synergetic and complementary for each other. It is important to remember the fact that some transcriptional elements may regulate several metabolic pathways and one metabolic pathway may need orchestrated regulation from some transcriptional elements, which is the nature of plant gene regulatory network system [100-115]. So, in some cases, only introducing a transcriptional element can not obtain targeted phenotype and may lead to metabolic unbalance in plants. In addition, because of coordinated evolution of transcriptional elements and their regulating metabolic pathways the genetically-modifying strategy for the same transcriptional element could produce different phenotypes in different plant species. Besides, some transcriptional elements not only regulate metabolic pathways, but also influence transport and allocation of secondary metabolites. Plant secondary metabolism plays an important role in plant responding to environmental stresses. Long-step progress has taken place in terms of introducing transcriptional elements to regulate targeted pathways. These issues need deeper exploration to establish an efficient genetically-modifying system by transcriptional elements and their network system for improving plant stress resistance and global eco- environment and feeding the world [53-63]. Potential genes mediating resistance to soil water stress and related abiotic stress in plants have been listed in Table **2** for reference.

The plant stress resistance mainly depends on varying proteins directly. There are many kinds of anti-proteins upon water stress, but we must point out that, in evolutionary terms, the possibility of producing specific proteins normally is very low except seriously drought-stress. Changing the relative content of different proteins in plants is the main way for resisting the stress, which is reflected in many plants such as NADP-malic enzymes in wheat and rice. It has an internal molecular basic of the drought-resistance in the drought-resistance species [127-130].

## 6. CONCLUDING REMARKS AND PERSPECTIVES

Plants have more refine mechanisms to regulate themselves from molecular level to ecosystem to respond to environmental changing. For instance, there are many coding-protein genes downstream only for osmotic regulation in abiotic stress resistance (Table **3**). Plants are always in the state of passiveness for confronting environmental succession and the related issue is more complicated, which is the main cause that plants are behind animals in the study of most fields [52-56].

Charting plant gene regulatory network system under soil water deficits is a great challenge. Nowadays, there are indeed many favorable conditions for charting this blueprint, including much available data from *Arabidopsis*, rice, grass, yeast and fruit fly, but the range of tested plants is very much limited, many stress-responsive genes have not been unified in terms of their refine functions, and many genes participating in environmental stresses are interacted and overlapped, which have led to incorrect placing of key genes (gene effectors) and signal molecules in the whole plant gene regulatory network system. Besides, much data are from under condition of one type of stresses. It is a fact that plants always confront more than two kinds of individual environmental stresses or their combination simultaneously in field [91-115]. Although drawing this dimensional plant gene regulatory network system with great details and complete pathways is impossible currently, the basic draft for this blueprint could be summarized in Fig. (**2**). This draft was established in combination with recent advance in this hot topic and from the context of development, which will provide instructions for further investigation and insights into understanding of plant refine plasticity for abiotic environmental stresses.

In a word, precise elucidation of plant gene regulatory network system under abiotic stresses is of importance to molecularly engineering plant resistance, because of which many excellent scientists world-wide have been engaged in this frontier field, resulting in a long-step progress. There are also many issues remained to be solved and needed to make efforts. Scope of tested plants needs to be extended; comprehensive study on a combination of environmental stress factors in laboratories and in field should be given much attention; system development viewpoint and computer simulation analysis method should be also applied. The combination of molecular biology, biotechnology and plant physiology (especially in field) is also the key. With accumulation of data from being extended plant range, plant gene regulatory network system under environmental stresses will be clearer and clearer.

## Figures and Tables

**Fig. (2):**
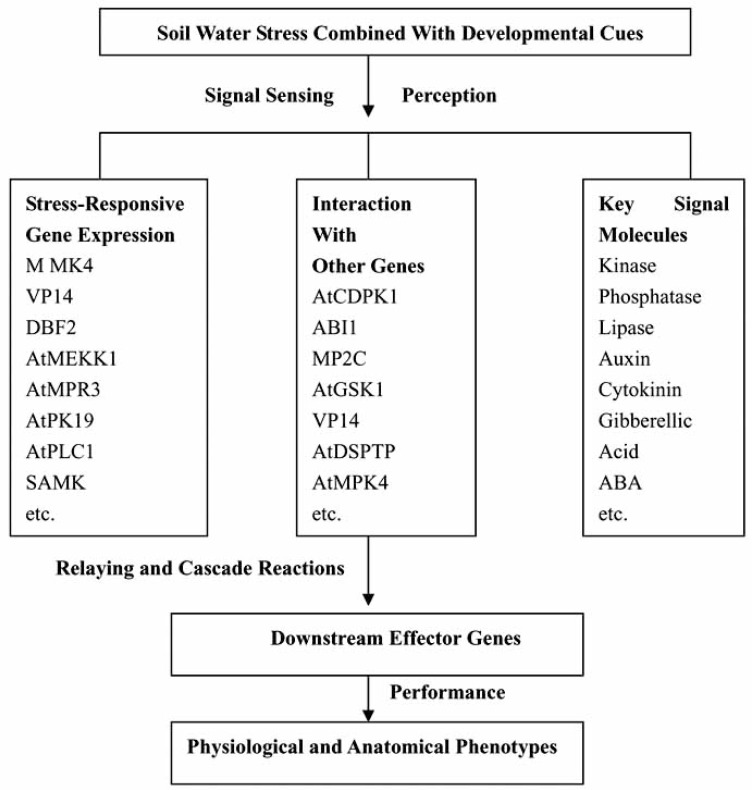
The basic draft for plant gene regulatory network system.

**Table 1.** Typical Transcriptional Elements Related to Abiotic Stresses in Plants and Crops.

Plant Materials	Factors	Binding Sites/Factor Types
*Arabidopsis thaliana*	ABI5/AtDPBF	ABA response elements(ABREs)/bZIP
*A.thaliana *	AtDPBF2	ABA response elements(ABREs)/bZIP
*A.thaliana*	AtDPBF3/AREB3	ABA response elements(ABREs)/bZIP
*A.thaliana*	AtDPBF4	ABA response elements(ABREs)/bZIP
*A.thaliana*	AtDPBF5/ABF3	ABA response elements(ABREs)/bZIP
*A.thaliana*	ABF1	ABA response elements(ABREs)/bZIP
*A.thaliana*	ABF2/AREB5	ABA response elements(ABREs)/bZIP
*A.thaliana *	ABF4/AREB2	ABA response elements(ABREs)/bZIP
*A.thaliana*	GBF3	ABA response elements(ABREs)/bZIP
*A.thaliana *	AB53	RY/sph elements/B3 domain proteins
*A.thaliana *	ATMTB2	MTC
*A.thaliana*	ATHB6	HD-Zip
*A.thaliana *	ATHB7	HD-Zip
*A.thaliana *	ATHB12	HD-Zip
*A.thaliana *	ABI4	AP2
*Oryza *	TRAB1	ABA response elements(ABREs)/bZIP
*Oryza*	OsVPI	RY/sph elements/B3 domain proteins
*Zea mays*	VP1	MYB
*Triticum *	EmBP-1	ABA response elements(ABREs)/bZIP
*Avena *	AtVPI	RY/sph elements/B3 domain proteins
*Helianthus *	DPBF5,-2,-3	ABA response elements(ABREs)/Bzip
*Phaseolus *	ROM2(repressor)	ABA response elements(ABREs)/Bzip
*Phaseolus *	PIARF	RY/sph elements/B3 domain proteins
*Craterestinma*	Cpvp1	RY/sph elements/B3 domain proteins
*Daucus *	C-ABI3	RY/sph elements/B3 domain proteins
*Populus *	PtABI3	RY/sph elements/B3 domain proteins

**Table 2.** Potential Genes Mediating Resistance to Soil Water Stress and Related Abiotic Stress in Plants

Gene	Gene Action	Species	Phenotype
*adc*	Polyamine synthesis	Rice	Drought resistance
*Apo-Inv *	Apoplastic invertase	Tobacco	Salt tolerance, high “osmotic pressure”
*AtGolS2 *	Galactinol and raffinose accumulation	Arabidopsis	Reduced transpiration
*AtHAL3*	Phosphoprotein phosphatase	Tobacco	Improved salt, osmotic and Lithium tolerance of cell cultures
*AtHAL3a *	Phosphoprotein phosphatase	Arabidopsis	Regulate salinity and osmotic tolerance and plant growth
*ATP-PRT*	ATP-phosphoribosyltransferase	Alyssum	His accumulation and Nickel tolerance
*AtTPS1*	trehalose-6-phosphate synthase	Tobacco	Drought resistance; sustained photosyntehsis
*BADH-1 *	Betaine aldehyde dehydrogenase	Carrot	Salinity tolerance
*BADH-1 *	Betaine aldehyde dehydrogenase	Rice	Cd tolerance
*BADH-1*	Betaine aldehyde dehydrogenase	Maize	Salinity tolerance
*BADH-1*	Betaine aldehyde dehydrogenase	Tobacco	Heat tolerance in photosynthesis
*BADH-1*	Betaine aldehyde dehydrogenase	Tobacco	Salinity tolerance
*BADH-1 *	Betaine aldehyde dehydrogenase	Tomato	Maintenance of osmotic potential
*betA *	Choline dehydrogenase (glycinebetaine synthesis)	Maize	Drought resistance at seedling stage and high yieldafter drought
*betA *	Choline dehydrogenase (glycinebetaine synthesis)	Tobacco	Increased tolerance to salinity stress
*CHIT33, CHIT42*	Endochitinase synthesis	Tobacco	Salt and metal toxicity resistance (& disease)
*CMO*	Choline monooxygenase (glycine betaine synthesis)	Tobacco	Better in vito growth under salinity and osmotic (PEG6000) stress
codA	Choline oxidase (glycine betaine synthesis)	Arabidopsis	Increased stress tolerance
codA	Choline oxidase (glycine betaine synthesis)	Arabidopsis	Salt tolerance in terms of reproduction
*codA *	Choline oxidase (glycine betaine synthesis)	Arabidopsis	Seedlings tolerant to salinity stress and increasedgermination under cold
*codA *	Choline oxidase (glycine betaine synthesis)	Brassica juncea	Tolerance to stress induced photoinhibition
*codA *	Choline oxidase (glycine betaine synthesis)	Rice	Increased tolerance to salinity and cold
*codA *	Choline oxidase (glycine betaine synthesis)	Rice	Recovery from a week long salt stress
*codA *	Choline oxidase (glycine betaine synthesis)	Tobacco	Freezing toleance
*codA *	Choline oxidase (glycine betaine synthesis)	Tomato	Chilling tolerance in yield and oxidative stress tolerance
*codA *	Choline oxidase (glycine betaine synthesis)	Tomato	Chilling tolerance
*COR15a *	Cold induced gene	Arabidopsis	Increased freezing tolerance
*COX*	Choline oxidase (glycine betaine synthesis)	Rice	Salt and 'stress' tolerance
*Ect A...ect C*	Edtoin accumulation in chloroplasts	Tobacco	Salt and cold tolerance
*GS2 *	Chloroplastic glutamine synthetase	Rice	Increased salinity resistance and chilling tolerance
*IMT1 *	Myo-inositol o-methyltransferase (D-ononitol synthesis)	Tobacco	Better CO2 fixation under salinity stress. Better recovery after drought stress.
*LWR1, LWR2*	Solute accumulation (proline)	Arabidopsis	Growth, osmotic adjustment, water status
*M6PR*	Mannose-6-phosphate reductase	Arabidopsis	Mannitol accumulation under salt stress leading tosalt tolerance
*M6PR*	Mannose-6-phosphate reductase	Arabidopsis	Mannitol accumulation and salt tolerance due to chloroplast protection
*mt1D *	Mannitol-1-phosphate dehydrogenase (mannitol synthesis)	Arabidopsis	Increased germination under salinity stress
*mt1D *	Mannitol-1-phosphate dehydrogenase (mannitol synthesis)	Petunia	Chilling tolerance
*mt1D *	Mannitol-1-phosphate dehydrogenase (mannitol synthesis)	Tobacco	Increased plant height and fresh weight under salinity stress
*mt1D *	Mannitol-1-phosphate dehydrogenase (mannitol synthesis)	Tobacco	No contribution to sustained growth under salinityand drought stress.
*mt1D *	Mannitol-1-phosphate dehydrogenase (mannitol synthesis)	Wheat	Drought and salinity tolerance of calli and plants
*mt1D & GutD*	Mannitol-1-phosphate dehydrogenase & glucitol-6-phosphate dehydrogenase	loblolly pine	High salt tolerance due to mannitol and glucitol accumulation
*mtlD*	Mannitol-1-phosphate dehydrogenase (mannitol synthesis)	Populus tomentosa	Salinity tolerance
*Osm1 …Osm4 *	Osmotin protein accumulation	Tobacco	Drought and salt tolerance in plant water status andproline accumulation
*Osm1 ...Osm4 *	Osmotin protein accumulation	Strawberry	Proline accumulation & salt tolerance
*Osmyb4*	Cold induced transcription factor	Arabidopsis	Accumulation of compatible solutes
*Osmyb4*	Specifically cold inducible	Tobacco	Freezing and Chilling tolerance
*Osmyb4*	Cold induced transcription factor	Tomato	Drought but not cold resistance
*OsP5CS2*	Highly homologous to P5CS	Rice	Cold and salinity tolerance
*otsA *	Trehalose-6-phosphate synthase (trehalose synthesis)	Tobacco	Increased leaf dry weight and photosynthetic activityunder drought. Increased carbohydrate accumulation.
*otsB *	Trehalose-6-phosphate synthase (trehalose synthesis)	Tobacco	Increased leaf dry weight and photosynthetic activityunder drought. Increased carbohydrate accumulation.
*P5CR*	Pyrroline carboxylate reductase (proline accumulation)	Soybean	Antioxidants activity under stress
*P5CR*	Pyrroline carboxylate reductase (proline accumulation)	Soybean	Amino acid accumulation
*P5CS *	Pyrroline carboxylate synthase (proline synthesis) (tomato)	Citrus	Osmotic adjustment and drought resistance
*P5CS*	Pyrroline carboxylate synthase (proline synthesis)	Petunia	Drought resistance and high proline
*P5CS*	Pyrroline carboxylate synthase (proline synthesis)	Potato	Salinity tolerance
*P5CS*	Pyrroline carboxylate synthase (proline synthesis)	Rice	Increased biomass production under drought and salinity stress
*P5CS*	Pyrroline carboxylate synthase (proline synthesis)	Rice	Reduced oxidative stress under osmotic stress
*P5CS*	Pyrroline carboxylate synthase (proline synthesis)	Rice	Resistance to water and sainity stress
*P5CS*	Pyrroline carboxylate synthase (proline synthesis)	Soybean	Resistance to osmotic stress and heat
*P5CS*	Pyrroline carboxylate synthase (proline synthesis) (tomato)	Soybean	Drought resistance, high RWC, high proline
*P5CS*	Pyrroline carboxylate synthase (proline synthesis) (tomato)	Sugarcane	Drought resistance *via *antioxidant role of proline
*P5CS*	Pyrroline carboxylate synthase (proline synthesis)	Tobacco	Increased biomass production and enhance flower development under salinity stress
*P5CS*	Pyrroline carboxylate synthase (proline synthesis)	Tobacco	Freezing tolerance
*P5CS*	Pyrroline carboxylate synthase (proline synthesis)	Wheat	Drought resistance due to antioxidative action
*P5CS*	Pyrroline carboxylate synthase (proline synthesis) (tomato)	Yeast	Reduced growth under none-stress and some promoted growth under mild stress
*pdc1 *	Pyruvate decarboxylase overexpression	Rice	Increased submergence tolerance
*pdc1; pdc2*	Pyruvate decarboxylase overexpression	Arabidopsis	Hypoxic stress survival
*PPO*	Polyphenol oxidases suppression	Tomato	Drought resistance
*SAMDC*	S-adenosylmethioninedecarboxylase (polyamine synthesis)	Rice	Better seedling growth under a 2 day NaCl stress
*SAMDC*	S-adenosylmethioninedecarboxylase (polyamine synthesis)	Tobacco	drought, salinity, Verticillium and Fusarium wiltsresistance
*SMT*	selenocysteine methyltransferase	Arabidopsis, Indian Mustard	Selenium hyperaccumulation tolerance
*SPE*	Spermidine synthase	Arabidopsis	Chilling, freezing, salinity, drought hyperosmosis
*spe1-1; spe2-1*	Spermidine non-accumulating	Arabidopsis	Decreased salt tolerance
*SST/FFT*	Fructan accumulation	Potato	Reduced proline accumulation at low water status
*TaCRT*	Ca2+-binding protein	Tobacco	Better water status, WUE and membrane stability
*TPP1*	Trehalose synthesis	Rice	Salt and cold tolerance
*TPS; TPP*	Trehalose synthesis	Arabidopsis	Drought, freezing, salt and heat tolerance
*TPS1*	Trehalose synthesis	Tomato	Drought, salt and oxidative stress tolerance
*TPS1*	Trehalose synthesis	Potato	Delayed wilting under drought
*TPS1 & TPS2*	Trehalose synthesis	Tobacco	Maintenance of water status under drought stress
*TPSP*	Trehalose synthesis	Rice	Drought, salt and cold tolerance expressed by chlorophyll fluorescence
*WCOR15*	Cold induced gene	Tobacco	Increased freezing tolerance

**Table 3.** Some Examples of the Osmotic Regulating Genes Downstream in Abiotic Resistance

Components	Metabolic Functions	Gene/Proteins
ROS scavenging	Increase in ROS scavenging enzymes	GP, PHGPX
Chaperones	Heat-/cold-/salt-shock proteins; protein folding	
Hsp,Csp,Ssp,*DnaJ*		
Fructan	Osmoprotection	*SacB*
Trehalose	Osmoprotection	
*Tps;Tpp,*trehalase		
Glycine betaine	Protein protection and carbon sink	*codA*
Proline	Substrate for mitochondrial respiration; redox control	P5CS/P5CR
Ectoine	Osmoprotectant	*Ect*A,BC
K^+^-transporters	High affinity K^+^ uptake	Hkt1,Hak1
K^+^-channels	Low affinity or dual affinity K^+^ uptake	Akt1, Akt
H_2_O channel proteins	Membrane cycling control	TIP
